# Dynamic simulation of landscape ecological security and analysis of coupling coordination degree: A case study of Bole

**DOI:** 10.1371/journal.pone.0297860

**Published:** 2024-02-08

**Authors:** Lei Yao, Abudureheman Halike, Kaixuan Yao, Qianqian Wei, Hua Tang, Buweiayixiemu Tuheti, Jianmei Luo, Yuefan Duan

**Affiliations:** 1 College of Geography and Remote Sensing Sciences, Xinjiang University, Urumqi, China; 2 Xinjiang Key Laboratory of Oasis Ecology, Xinjiang University, Urumqi, China; 3 Key Laboratory of Smart City and Environment Modelling of Higher Education Institute, Xinjiang University, Urumqi, China; Jinan University, CHINA

## Abstract

The ecological security of oasis cities in arid and semi-arid regions is highly susceptible to changes in regional landscape patterns and the degree of coordination between human activities and the environment. At the same time, the ecological security of urban landscapes also profoundly affects the success of regional economic and environmental coordination and development. This study is based on land use data from 1990, 2000, 2010, and 2020, as well as land use data from the natural development scenario (NLD), economic development scenario (ECD), ecological development scenario (ELD) and ecological-economic development scenario (EED) simulated by the patch-generating land use simulation (PLUS) model in 2030. From the perspective of production-living-ecological land (PLEL), it analyzes the changes in the past and future landscape ecological security and coupling coordination characteristics of Bole. The results show that from 1990 to 2020, Bole was mainly dominated by grassland ecological land (GEL) and other ecological land (OEL), accounting for a total proportion of 69.51%, with a large increase in production and living land area; the average value of landscape ecological risk is decreasing, and the landscape ecological security of Bole is developing towards benignity; the area of highly coupled coordination zone is decreasing continuously, while that of basic coordination zone and moderate coordination zone is increasing continuously. Under the 2030 EED scenario, the overall changes in various types of land use are not significant, and the average value of landscape ecological risk is the smallest, but it is higher than that in 2020 as a whole; under EED scenario, the area of highly coordinated zone and moderate coordinated zone is the largest among four scenarios, and the best coupling coordination level among the four scenarios. Landscape ecological security and its coupling coordination will be affected by land use patterns. Optimizing regional land use patterns is of great significance for improving urban landscape ecological security and sustainable high-quality development.

## Introduction

In recent years, ecological security has become a hot topic among scholars as part of national security [1, 2]. It is also an important indicator for assessing the safety and health of regional ecosystems [3]. Urbanization, while driving economic and social development, also has significant impacts on ecosystems that cannot be ignored [4]. In this process, due to increasing human demand for land resources and the desire for transformation, the relationship between humans and the environment has become increasingly tense [5]. Climate change, environmental degradation, energy crises, and food shortages have intensified the contradictions between humans and the environment [6–9], resulting in varying degrees of changes in the spatial and temporal patterns of urban landscape ecological security [10]. This has led to structural fragmentation, functional impairment, and even a decline in ecosystem services, affecting human well-being and the sustainable development of society and the economy [4], as well as reducing urban resilience [11] and significantly increasing urban ecological risks. Changes in urban landscape patterns are not only influenced by human socioeconomic activities but also constrained by natural factors. Therefore, conducting a comprehensive evaluation of urban landscape ecological security under the rapid urbanization context and resolving the conflicts between humans and nature has become an important topic in the field of urban landscape ecology research [12], playing a vital role in promoting high-quality economic development and sustainable ecological environment [13].

Currently, many scholars are evaluating the ecological security of landscapes from different perspectives. The evaluation of landscape ecological security mainly focuses on urban areas, watersheds, wetlands, and lakes [14–17]. The research methods primarily include risk-based and pattern-based evaluation methods [18]. The evaluation scales mainly consist of grids, administrative units, and economic zones, while the research indicators primarily include fragmentation, dominance, separation, fractal dimension, diversity, etc. [19]. The research content generally includes ecological security assessment and ecological security patterns [20]. Currently, the use of GIS grid methods for selecting and constructing optimal granularity units has become a new approach for landscape ecological risk evaluation [21]. With its powerful spatial information mining and mapping capabilities, GIS can express land use information and landscape ecological security in spatial terms, effectively revealing the spatial patterns and spatial relationships of geographical features or phenomena [22]. It is worth noting that the introduction of the concept of "PLEL" (production-living-ecological land) to evaluate landscape ecological security is a meaningful exploration [23, 24]. Since the concept of PLEL was proposed at the 18th National Congress of the Communist Party of China [25], many scholars have conducted extensive research on it. The classification of land use status has been combined to divide the PLEL space [26, 27]. Evaluating the landscape ecological risks or security of regions from the perspective of PLEL has become a major focus of research [3, 20, 28].

With the development of landscape ecology, land-based landscape ecological security analysis has gradually become an important component of ecological security assessment [29]. Land not only serves as a macro representation of surface landscapes [18], but also as a fundamental material carrier for human production and livelihood [30, 31]. As the basis for integrating multiple surface processes and the main response to human-environment interactions [32], land use plays a decisive role in regional ecological security [33]. Within human-dominated landscapes, changes in land use patterns and intensity will have regional and cumulative impacts on ecosystems [23]. In recent years, with the rapid development of computer and geographic information technologies, land-based landscape ecological security assessment and simulation studies have become hot topics [34, 35]. Cai et al. explored the dynamic changes in landscape patterns and ecosystem service values under a low-carbon scenario [36], while Zhang et al. simulated and predicted landscape ecological risks and their coupling with ecosystem service functions [37, 38]. Various methods and models have been developed to predict future land use, such as the cellular automata (CA) and Markov model [39], conversion of land use and its effects at small regional extent (CLUE-S) model [40], future land use simulation (FLUS) model [41], and PLUS model [42], among others. Studies have shown that the patch-generating land use simulation (PLUS) model, which combines land expansion analysis strategies and a CA model based on multi-type random patch seeds, achieves higher simulation accuracy and more equivalent landscape patterns [43], proving its advantages in simulating real landscapes, including high precision and fast processing speed in simulating land use change [5, 42, 44]. Therefore, the PLUS model has wide applicability in multi-scenario simulation of land use, as well as in the simulation of ecosystem services and landscape ecological risks [13, 45, 46]. In terms of calculating future land demand under different scenarios, the gray multi-objective programming (GMOP) model, by coupling the gray prediction model GM(1,1) and multi-objective programming model (MOP) [13], can fully consider the uncertainty of objective functions and constraints in future land use, and provide decision-makers with optimal land use optimization and allocation plans using a multi-constraint solving approach for multi-objective problems [47]. At the same time, the combination of the PLUS model and GMOP model can leverage their advantages in predicting land use quantity and spatial distribution, achieving dual simulation of regional land use changes in quantity and space [48].

Due to the relatively fragile natural environment of oasis cities in arid and semi-arid regions, their landscape ecological security is easily disturbed by external environmental factors. Since the 1950s, due to large-scale water and soil development, most of the rivers that used to flow into Ebinur Lake have been cut off, resulting in a sharp decrease in the lake’s water volume. In 2019, the ecological environment water consumption accounted for only 3.1% of the total water consumption, and the contradiction between local social economy and ecological environment water use is increasing [49], which has seriously affected the sustainable development of the local and regional ecological environment. As an important part of the Ebinur Lake Basin and an important ecological barrier, Bole has attracted attention to its landscape ecological security from outside. Bole is not only the capital of Bortala Mongolian Autonomous Prefecture and the political, economic, and cultural center of the whole prefecture, but also an important member of China’s Silk Road Economic Belt core area and Xinjiang Tianshan North Slope Economic Belt. It is also an important part of Ebinur Lake Basin, a typical oasis city in northwest China’s arid region. Therefore, Bole has great potential for future development and undertakes comprehensive tasks such as economic construction, social development, and ecological environment protection. At the same time, with the proposal of national ecological civilization construction, the implementation of the concept that "green mountains and clear waters are gold and silver mountains", and the need to adhere to integrated protection and systematic management of mountains, waters, forests, fields, lakes, grasses, sands, land use changes have gradually increased their impact on ecosystems [3]. Therefore, simulating changes in land use and landscape ecological security in Bole is conducive to optimizing land use patterns, improving landscape ecological security and promoting benign development of ecosystems.

To provide decision support for land use management of oasis cities in arid and semi-arid areas, and to provide a theoretical reference for landscape ecological security, ecological environment protection, and resource development and utilization, this study explored the landscape ecological security of Bole from 1990 to 2020 and its spatial coupling evolution characteristics based on the PLEL perspective. The PLUS model was used to simulate the spatial coupling relationship between landscape ecological security features and various elements in different scenarios in 2030. Firstly, the spatiotemporal changes of PLEL in Bole from 1990 to 2020 were analyzed. Then, the distribution of PLEL under multiple scenarios in 2030 was analyzed. Finally, the changes and coupling characteristics of landscape ecological security in the past and under multiple scenarios were analyzed separately.

## Study area overview and data sources

### The study area

**Fig 1:**
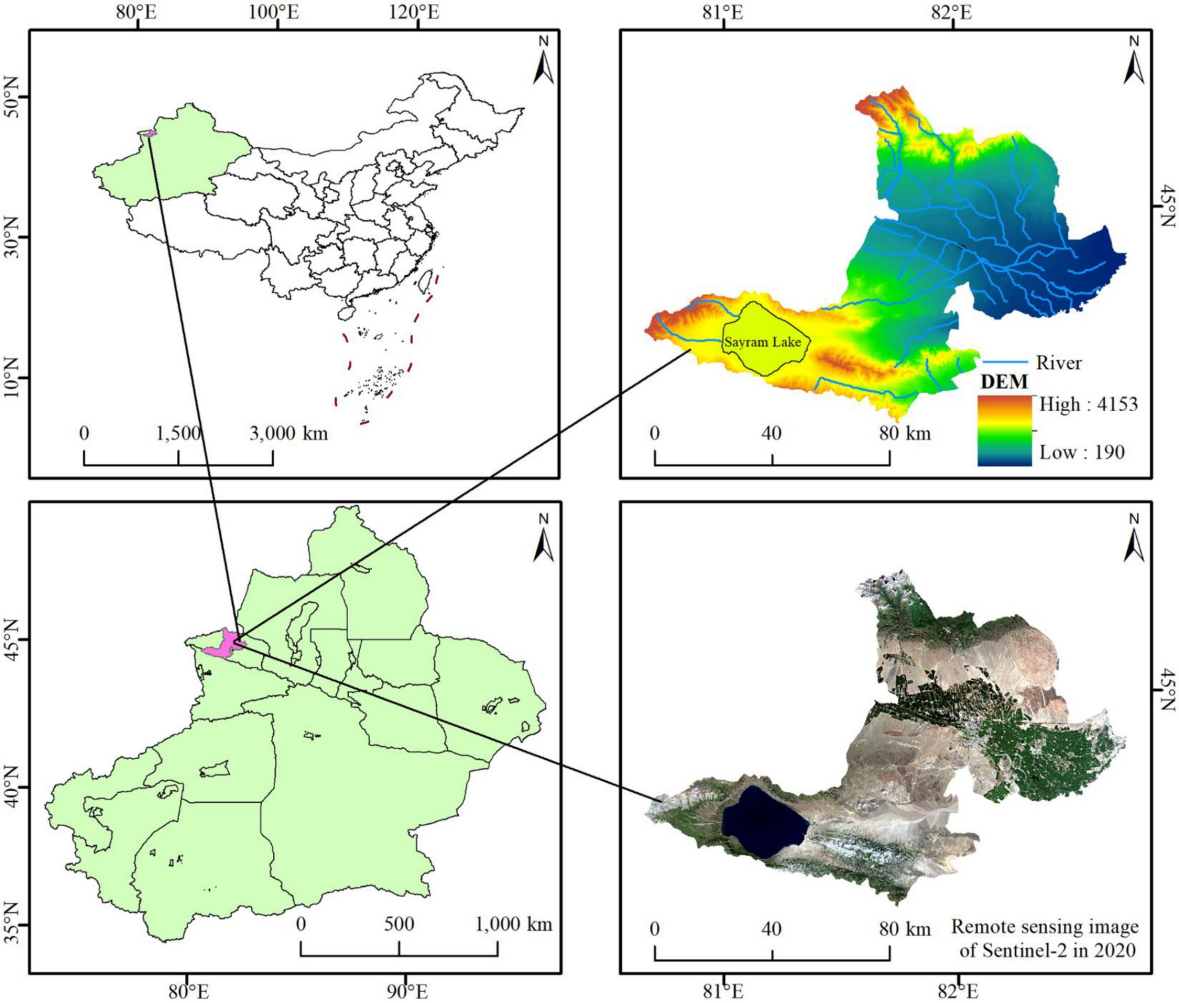
Overview map of the research area. Republished from [52] under a CC BY license, with permission from Resource and environmental science data registration and publishing system, 2023.

Bole (80°39’E-82°44’E, 44°20’N-45°23’N, including Shuanghe of the Corps) is located in the northwest of Xinjiang, China (Fig 1). It is a county-level city under the jurisdiction of Bortala Mongolian Autonomous Prefecture in Xinjiang Uygur Autonomous Region. It has 4 towns, 1 township, 1 state-owned ranch and 5 streets. The terrain within the territory is high in the west and low in the east, including mountains, deserts and oases. It belongs to the typical region of mountain-oasis-desert system. The land is distributed vertically and presents the topography of “four mountains, three valleys and two rivers”. The Bortala River runs through the middle from west to east, and the oasis is distributed along the river in a strip [50]. The temperature in Bole varies greatly in spring, hot in summer, less rain in autumn and cold in winter. The annual average temperature is 6.8°C and the annual average precipitation is 200.3mm. It belongs to the continental temperate desert arid climate. In 2020, Bole had a total population (POP) of 260,300 people and achieved a regional gross domestic product (GDP) of RMB 17.41 billion. The economic development speed of Bole is accelerating, and the urban functions and living environment are constantly improving, which will inevitably have a certain impact on the local ecosystem, functions, security, etc. [51].

### Data sources and methods

The land use data is sourced from the Resource and Environment Science and Data Center (RESDC) of the Chinese Academy of Sciences, in 1990, 2000, 2010, 2015 and 2020, with a spatial resolution of 30 m × 30 m. The overall kappa coefficient of the data is over 90% [53]. The social and economic data, POP, DEM and land use driving factors used in this paper are shown in Table 1. To ensure consistency in spatial resolution, coordinate system, and other aspects of the data, the coordinate transformation, mask extraction and resampling tools in ArcGIS are used to process the driving factors and land use data. Unified spatial resolution of 30 m× 30 m, unified coordinate system WGS_ 1984_ UTM_ Zone_ 44N.

**Table 1:** Data sources.

Data type	Data name	Data source
Land use data	Land use data for 1990, 2000, 2010, 2015 and 2020	RESDC (https://www.resdc.cn )
Natural factor data	DEM, Aspect, Slope	NASA SRTM DEM, aspect and slope are calculated according to DEM (https://www.nasa.gov/ ).
	Precipitation, Temperature	National Data Center for Earth System Science (https://www.geodata.cn ).
	Aridity, Erosion, Soil	Center for Resources and Environmental Sciences and Data, Chinese Academy of Sciences (https://www.resdc.cn ).
Road and river data	Main roads, primary roads, secondary roads, highway and rail networks, water systems	National fundamental geographic information center at (www.ngcc.cn/ngcc/html/1/396/397/16118.html).
Economic and demographic data	GDP、POP	RESDC (https://www.resdc.cn )

This study divides PLEL into 8 secondary categories according to the land use classification system (Table 2), namely: agricultural production land (APL), industrial production land (IPL), urban living land (ULL), rural living land (RLL), forest ecological land (FEL), grassland ecological land (GEL), water ecological land (WEL) and other ecological land (OEL).

**Table 2:** Classification of dominant functions of land use.

First level land use type	Secondary land use types	Three-level classification
Productive land	APL	Paddy field, dry land
	IPL	Industrial and mining construction land
Living land	ULL	Urban residential land
	RLL	Rural residential area
Ecological land	FEL	Woodland, shrub land, dredge land, other woodland
	GEL	High coverage grassland, medium coverage grassland, low coverage grassland
	WEL	Canals, lakes, reservoirs, permanent glaciers, tidal flats, tidal flats
	OEL	Sandy land, Gobi, saline-alkali land, marshland, bare land, bare rock gravel land

### Data sources and methods

**Fig 2:**
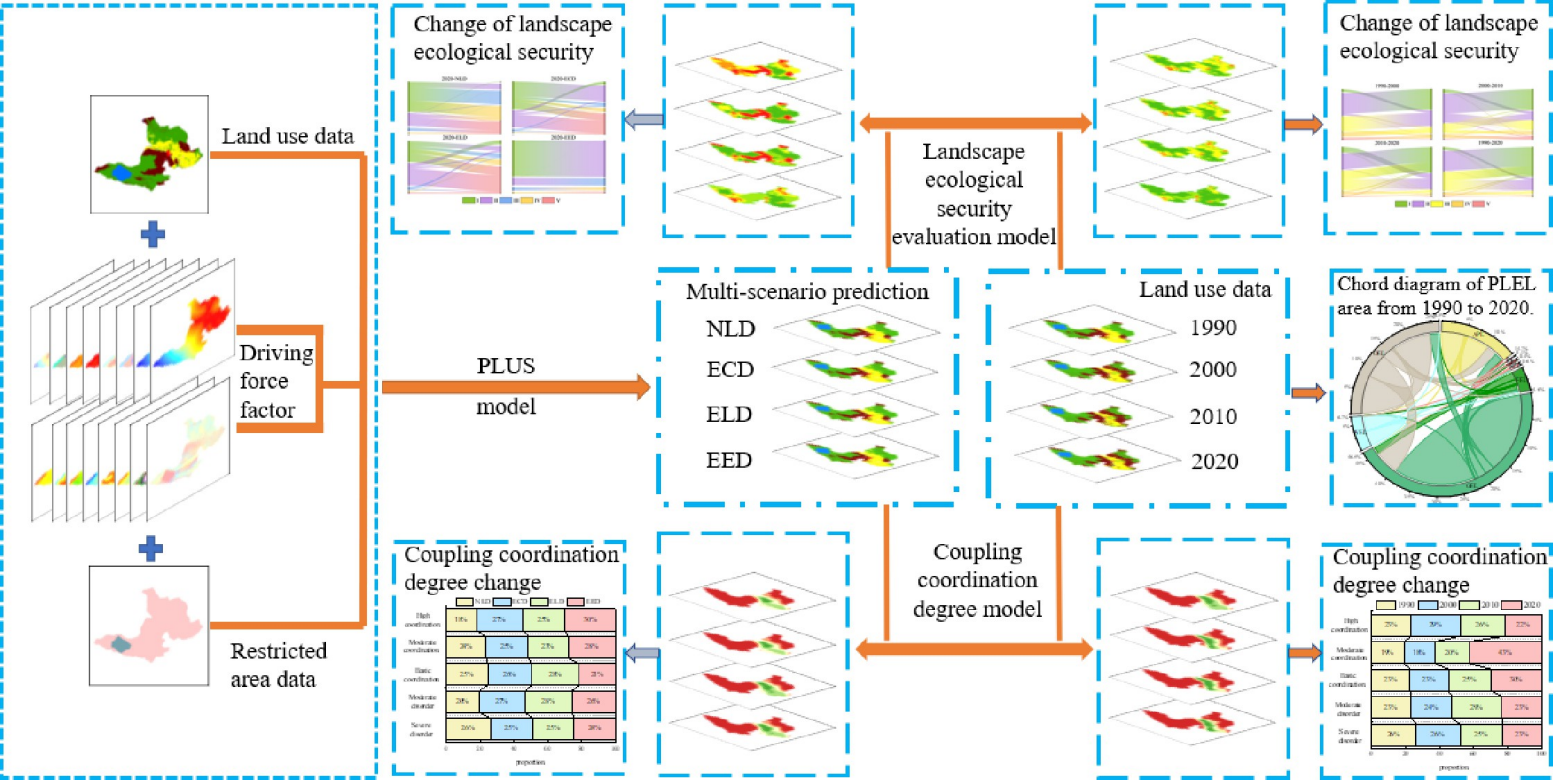
Overview map of the research area.

Fig 2 shows the workflow of this study. Firstly, the natural development scenario (NLD), economic development scenario (ECD), ecological development scenario (ELD) and ecological-economic development scenario (EED) are predicted by inputting land use data, driving factors and restriction area data into the PLUS model. Then analyze the changes in land use, landscape ecological security, and PLEL coupling co scheduling for the four scenarios predicted from 1990 to 2020.

#### Landscape ecological security analysis

Based on previous studies [54], we divided the study area into 855 grids of 3 km × 3 km using ArcGIS 10.8 software as evaluation units. Then, Fragstats software was used to calculate landscape pattern indices for each evaluation unit. Finally, landscape fragmentation, isolation, dominance, as well as disturbance and vulnerability, were selected to construct the Landscape Ecological Risk Index (ERI) model.


$$ERI_{i}=\sum_{i=1}^{N}\frac{A_{k_{i}}}{A_{k}}R_{i}$$ (1)

$$R_{i}=E_{i}\times F_{i}$$ (2)

$$E_{i}=aC_{i}+bN_{i}+cD_{i}$$ (3)

$$C_{i}=\frac{N_{i}}{A_{i}},N_{i}=\frac{A}{2A_{i}}\times \sqrt{\frac{n_{i}}{A}},D_{i}=\frac{Q_{i}+M_{i}}{4}+\frac{L_{i}}{2}$$ (4)

In the formula: *ERI*_*i*_ is the landscape ecological risk index of the i-th evaluation unit, $A_{k_{i}}$ is the area of the i-th type of landscape in the k-th evaluation unit, *A*_*k*_ is the area of the k-th evaluation unit, and *R*_*i*_ is the landscape loss index of the i-th type of landscape. *N*_*i*_ is the number of patches of landscape type i, *A*_*i*_ is the total area of landscape type i, *A* is the total area of landscape; *Q*_*i*_ is the number of sample plots where patch i appears/total number of sample plots, *M*_*i*_ is the number of patches i/total number of patches, *L*_*i*_ is the area of patch i/total area of sample plots. *a*, *b* and *c* are weights for *C*_*i*_, *N*_*i*_ and *D*_*i*_ respectively, and *a* + *b* + *c* = 1. According to relevant references [55] and expert opinions, a weight value of 0.5, 0.3 and 0.2 was assigned to *a*, *b* and *c* respectively. The eight secondary land use categories were assigned values from low to high [3]: ULL = 1, RLL = 2, FEL = 3, GEL = 4, APL = 5, WEL = 6, IPL = 7, and OEL = 8. After normalization, their sizes are respectively: 0.03, 0.06, 0.08, 0.11, 0.14, 0.17, 0.19 and 0.22.

#### Construction of coupling coordination degree model

The PLEL can be classified into three types: production land, living land, and ecological land on the first level. These three types of land use interact and constrain each other spatially. In order to better reflect the coupling and coordination characteristics of PLEL in the study area, a coupling coordination degree model is adopted to reveal the spatiotemporal evolution characteristics of the coupling coordination degree among the three types of land use. The calculation formula is as follows [56]:

$$C=\sqrt{\left[1-\frac{\sqrt{{\left(U_{3}-U_{1}\right)}^{2}}+\sqrt{{\left(U_{3}-U_{2}\right)}^{2}}+\sqrt{{\left(U_{2}-U_{1}\right)}^{2}}}{3}\right]\times \sqrt{\frac{U_{1}}{U_{3}}\times \frac{U_{2}}{U_{3}}}}$$ (5)

$$T=\alpha_{1}U_{1}+\alpha_{2}U_{2}+\alpha_{3}U_{3}$$ (6)

$$D=\sqrt{C\times T}$$ (7)

In the formula: *C* is the coupling degree of the PLEL; *U*_1_, *U*_2_ and *U*_3_ are the ecological landscape risk values of production land, living land and production land respectively; *D* is the coupling coordination degree; *T* is the comprehensive evaluation value of ecological security; *α*_1_, *α*_2_, *α*_3_ are the weights of the three landscape ecological space securities. Referring to previous research results [20], they are assigned as follows: *α*_1_ = *α*_2_ = *α*_3_ = 0.3.

#### PLUS model and simulation process

The PLUS model is a software for predicting land use change, which mainly integrates two modules: the Land Expansion Analysis Strategy (LEAS) based module and the CA module based on multi type random patch seeds, and has high simulation accuracy [42].

Multi-scenario simulation: The GMOP model is composed of the gray prediction model GM (1,1) and the multi-objective programming model (MOP). The model fully considers the uncertainty of the objective function of future land use types. The target scenarios and constraint conditions are set as shown in Tables 3 and 4. The LIN-GO11 software and the genetic algorithm module in MATLAB are used to solve the multi-objective function, and finally the demand for land use under different scenarios in the future is obtained.

**Table 3:** Target scenario setting.

Scenarios	Objective function	description
NLD	––	The Markov chain module in the PLUS model is used to calculate the land demand in Bole in 2030.
ECD	Max*F*_1_(*x*) = 2.53*x_1_+278.26*(*x*_2_+*x*_3_+*x*_4_)+0.93*x_5_+0.51*x_6_+0.007*x_7_+0*x_8_	Maximize economic benefits, where *x*_1-8_ are the areas of APL, IPL, ULL, RLL, FEL, GEL, WEL and OEL, respectively, with a unit of ten thousand yuan per hectare. The LINGO11 software is used.
ELD	Max*F*_2_(*x*) = 0.9*x_1_+0*(*x*_2_+*x*_3_+*x*_4_)+3.93*x_5_+2.63*x_6_+19.95*x_7_+0.16*x_8_	Maximize economic benefits, where *x*_1-8_ are the areas of APL and IPL, respectively, with a unit of ten thousand yuan per hectare. The genetic algorithm module in MATLAB software is used.
EED	Max {*F*_1_(*x*), *F*_2_(*x*)}	Balance ecological protection and economic development.

**Table 4:** Constraints and descriptions.

Constraint factor	Constraint condition(hm²)	explanation
Total land area constraint	*x*_1_+*x*_2_+*x*_3_+*x*_4_ +*x*_5_+*x*_6_+*x*_7_+*x*_8_ = 732360.78	The total area of land remains unchanged during the development process.
APL constraint	135559.17≤*x*_1_≤ 144917.1	Taking the cultivated land area in 2020 as the lower limit and the cultivated land area in the natural development state as the upper limit.
IPL constraint	2054.16≤*x*_2_≤ 3319.02	The built-up area in 2020 predicted by Markov chain is used *as* the lower limit and the built-up area in 2030 is used as the upper limit.
ULL constraint	4312.8≤*x*_3_≤ 4424.58	
RLL constraint	6737.22≤*x*_4_≤ 9719.19	
FEL constraint	20140.53≤*x*_5_≤22562.69	The minimum area of forestland is determined by the rate of degradation from 2015 to 2020, while the maximum area is 1.1 times the forestland area under the natural expansion scenario.
GEL constraint	353841.57≤*x*_6_≤357380.01	The minimum area of grassland is the grassland area under the natural development scenario, while the maximum area is 1.01 times the grassland area under the natural development scenario.
WEL constraint	48977.01 ≤*x*_7_≤ 49388.31	The water area of Bole is on the rise. The water area in 2020 is set as the lower limit, and the water area predicted by Markov chain in 2035 is used as the upper limit. Lake Sayram is a spatial restriction zone.
OEL constraint	*x*_8_≤151034.49	Taking the unused land area in 2020 as the upper limit to retain land supply capacity.
Total population constraint	0.006*x_1_+40*x_3_+1.3*x_4_+0.0015*x_5_+0.003*x_6_≤320000	The population density of APL is 0.006 people/hectare, the population density of ULL is 40 people/hectare, the population density of RLL is 1.3 people/hectare, the population density of FEL is 0.0015 people/hectare, and the population density of GEL is 0.003 people/hectare [5].

## Results

### Analysis of temporal and spatial changes of Bole PLEL

**Fig 3:**
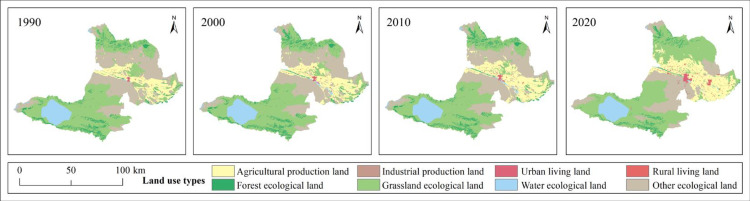
Spatial distribution pattern of PLEL from 1990 to 2020. Republished from [57] under a CC BY license, with permission from Resource and environmental science data registration and publishing system, 2023.

**Fig 4:**
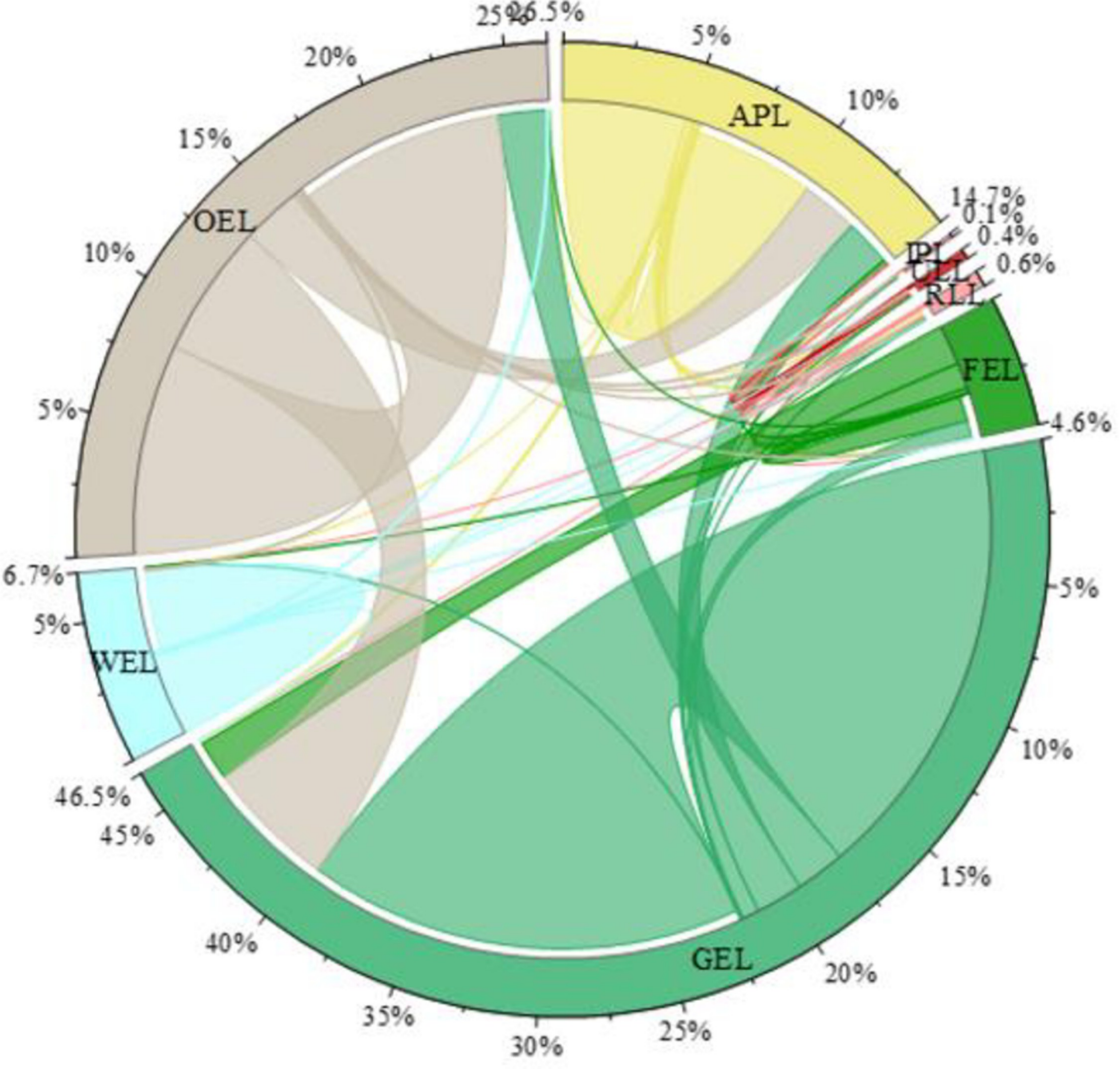
Chord diagram of PLEL area in Bole from 1990 to 2020.

According to Table 5, Figs 3 and 4, Bole is mainly composed of GEL and OEL. As of 2020, GEL accounts for 48.89% of the total area and OEL accounts for 20.62% of the total area. From 1990 to 2020, FEL and WEL showed an increasing trend first and then a decreasing trend, while OEL showed a decreasing trend. Among them, FEL decreased from 41066.28 hm² in 1990 to 25626.33hm² in 2020, a decrease of -37.60%, and was mostly transferred to GEL; WEL did not change much; OEL decreased from 236848.8hm² in 1990 to 151038.6 hm² in 2020, a decrease of -36.23%, with northern OEL being squeezed out by grassland and cultivated land, and southern OEL mainly being transferred from GEL. GEL showed a decreasing trend first and then an increasing trend, increasing from 322489.3hm² in 1990 to 358050.3hm² in 2020, an increase of 11.03%, mainly transferred from FEL and OEL. The increase in GEL is inseparable from climate change and ecological protection measures. Due to the needs of cultivated land reclamation and socio-economic development, FEL and OEL are occupied more by production and living land. Therefore, during this period, APL, IPL, ULL and RLL showed an overall increasing trend. Among them, APL increased from 79964.1hm²in 1990 to 135554.9hm² in 2020, an increase of 69.52%. APL expanded northward and was mainly transferred from GEL and OEL; IPL increased from 36.81hm² in 1990 to 2057.76hm² in 2020, an increase of 5490.22%; ULL increased from 1159.56hm² in 1990 to 4311.9hm² in 2020, an increase of 271.86%, mainly transferred from APL and OEL; RLL increased from 1558.89hm² in 1990 to 6735.42hm² in 2020, an increase of 332.07%, mainly transferred from APL. The significant increase in APL, IPL, ULL and RLL indicates that Bole has developed rapidly in industrialization, urbanization and land reclamation over the past thirty years.

**Table 5:** 1990–2020 bole PLEL area and change.

PLEL	Area (hm²)				Area change (hm²)			
	1990	2000	2010	2020	1990–2000	2000–2010	2010–2020	1990–2020
APL	79964.1	90171.72	109254.7	135554.9	10207.62	19082.98	26300.2	55590.8
IPL	36.81	36.63	278.1	2057.76	-0.18	241.47	1779.66	2020.95
ULL	1159.56	1594.26	1923.84	4311.9	434.7	329.58	2388.06	3152.34
RLL	1558.89	2192.31	2334.24	6735.42	633.42	141.93	4401.18	5176.53
FEL	41066.28	44919.36	45286.47	25626.33	3853.08	367.11	-19660.14	-15439.95
GEL	322489.3	308730.7	301611.2	358050.3	-13758.6	-7119.5	56439.1	35561
WEL	49025.88	51138.72	50909.31	48976.56	2112.84	-229.41	-1932.75	-49.32
OEL	236848.8	233164.1	220747.2	151038.6	-3684.7	-12416.9	-69708.6	-85810.2

In the past 30 years, the area of FEL and OEL has decreased, while the area of WEL and production and living land has increased.

### Analysis of land use simulation results under multiple scenarios

This study compared the simulated land use from 2015–2020 with the actual land use in 2020. The simulation resulted in a Kappa coefficient of 0.953 and an overall accuracy of 96.82%, indicating that the PLUS model is highly reliable. Thus, it can be utilized to simulate the land use scenario in Bole in 2030.

**Fig 5:**
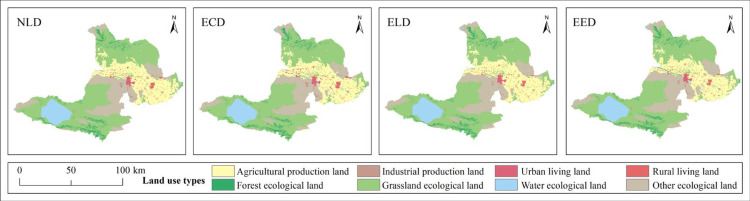
Spatial distribution pattern of PLEL under different scenarios in 2030. Republished from [57] under a CC BY license, with permission from Resource and environmental science data registration and publishing system, 2023.

The NLD scenario uses the Markov chain simulation of future land use demand based on the development trend from 2015 to 2020, combined with the PLUS model to simulate the natural development scenario. According to Table 6 and Fig 5, under this scenario, the area of APL, IPL, ULL and RLL increased, among which APL increased the most, with an increase of 9044.01hm². IPL had the largest increase rate of 61.29%; FEL, GEL, WEL and OEL decreased in area, among which GEL and OEL decreased the most, by 4208.76hm² and 4481.01hm² respectively. FEL had the largest decrease rate of -11.84%. The ECD scenario requires that the development of various land uses should ensure that economic benefits can be maximized. Therefore, the area of IPL and RLL has increased significantly, reaching 61.29% and 44.3% respectively, with corresponding ecological land sacrificing. Among them, the area of FEL, GEL and OEL has decreased. OEL has decreased by 10388.79 hm² with a decrease rate of -6.88%. The ELD scenario requires that the development of various land uses should ensure that ecological benefits can be maximized. The changes in area and change rate of IPL, ULL and RLL are extremely small. WEL and APL have increased in area, among which APL has increased the most by 7553.43hm², while other land use types have decreased slightly. The EED scenario takes into account both ecological protection and economic development, with little overall change in magnitude. Among them, IPL had the largest increase rate of only 11.36%, and ecological land was protected to some extent as a whole.

**Table 6:** PLEL area under different scenarios. Unit: hm².

year	Scenarios	APL	IPL	ULL	RLL	FEL	GEL	WEL	OEL
2020	—	135554.94	2057.76	4311.9	6735.42	25626.33	358050.33	48976.56	151038.63
2020–2030	NLD	144654.51	3301.5	4386.54	7643.25	22593.42	353841.57	49382.37	146557.62
	ECD	142773.75	3319.02	4424.58	9719.19	24706.08	357380.01	49388.31	140649.84
	ELD	143108.37	2054.16	4312.8	6737.22	24483.6	357317.43	49276.17	145071.03
	EED	135959.31	2291.49	4317.48	6834.15	25563.06	357244.65	49380.66	150769.98
2020–2030	NLD	9099.57	1243.74	74.64	907.83	-3032.91	-4208.76	405.81	-4481.01
	ECD	7218.81	1261.26	112.68	2983.77	-920.25	-670.32	411.75	-10388.79
	ELD	7553.43	-3.6	0.9	1.8	-1142.73	-732.9	299.61	-5967.6
	EED	404.37	233.73	5.58	98.73	-63.27	-805.68	404.1	-268.65

The land type changes in NLD, ECD, and ELD scenarios are relatively large, while the land type changes in EED scenarios are small.

### Analysis of landscape ecological security changes and coupling characteristics

#### Analysis of landscape ecological security changes

**Fig 6:**
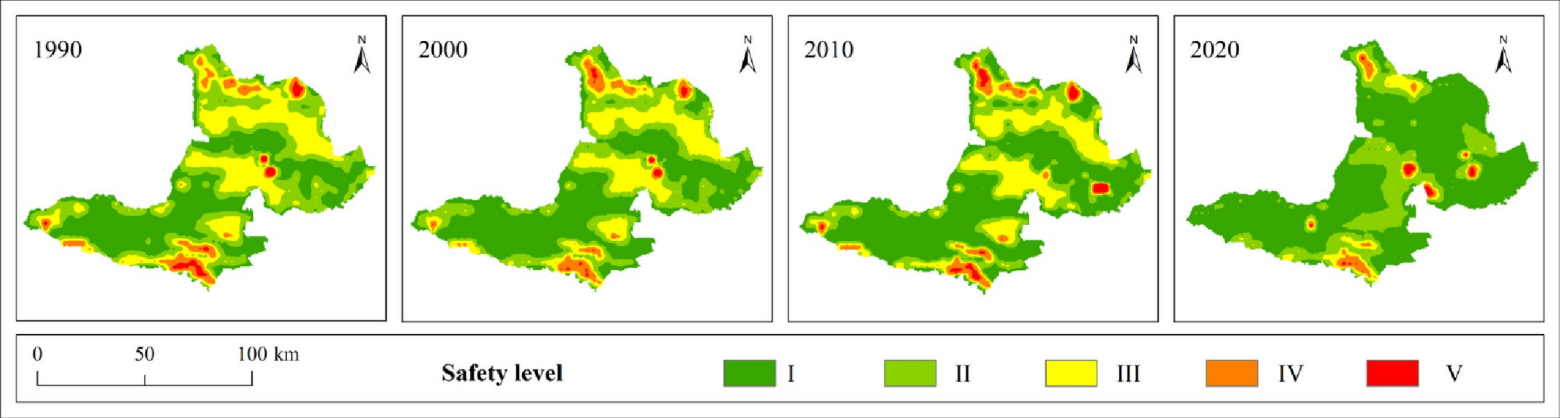
Spatial distribution pattern of Bole landscape ecological security level from 1990 to 2020. Republished from [57] under a CC BY license, with permission from Resource and environmental science data registration and publishing system, 2023.

**Fig 7:**
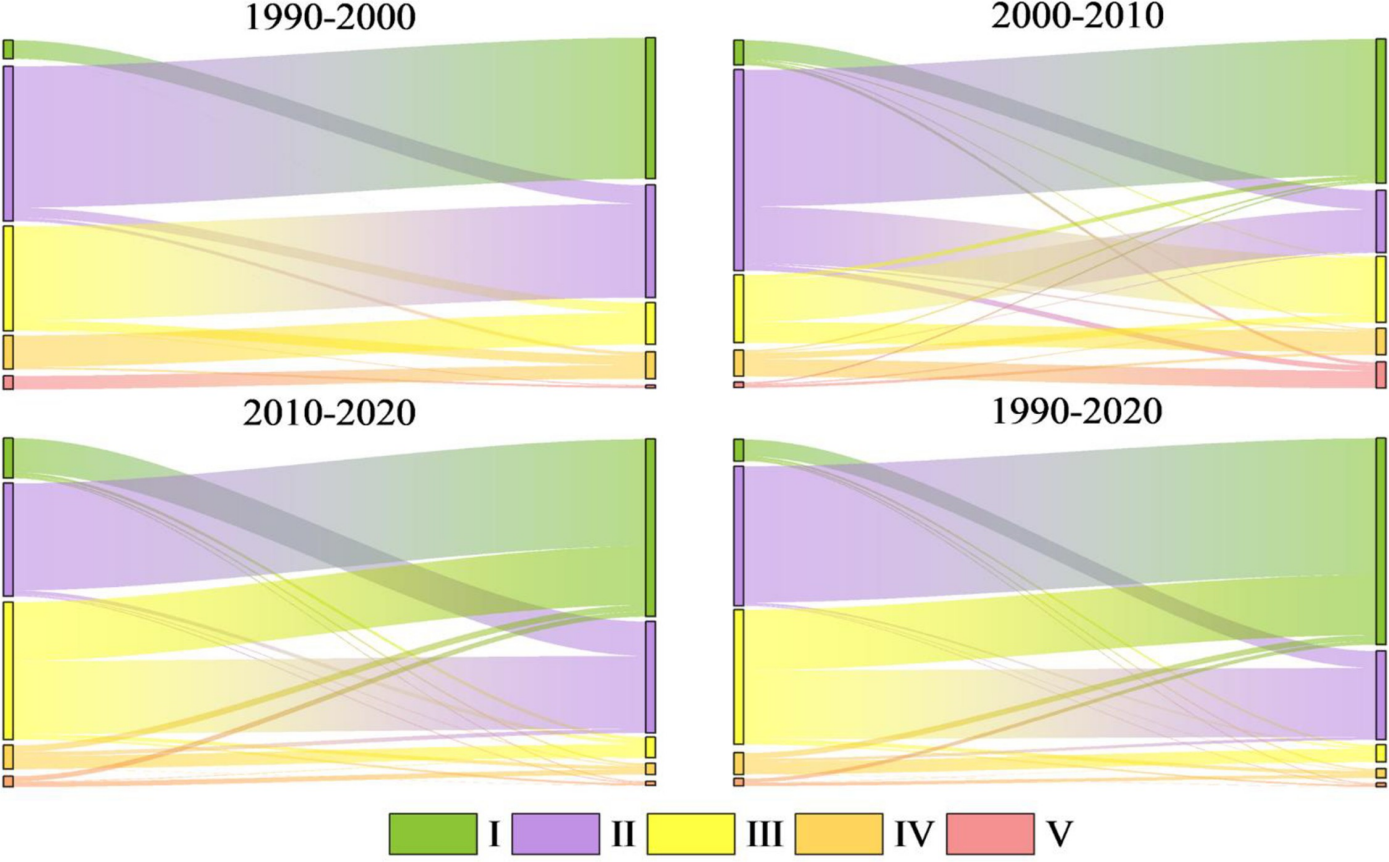
Sankey Map of landscape ecological security transfer from 1990 to 2020.

We explore the spatial and temporal distribution characteristics of landscape ecological security in Bole by assigning landscape ecological risk values to the central points of each evaluation unit grid. The ordinary Kriging method in the ArcGIS geostatistical module is used for spatial interpolation processing, and the natural breakpoint method and artificial fine-tuning threshold are combined to divide the research area in 2010 into 5 levels: safe zone I (ERI<0.0061), relatively safe zone II (0.0061≤ERI<0.0079), medium safe zone III (0.0079≤ERI<0.0096), relatively unsafe zone IV (0.0096≤ERI<0.012), and unsafe zone V (ERI≥0.012). The ecological security classification of other years is based on the classification interval of 2010 for comparison between data in different periods, as shown in Table 7, Figs 6 and 7.

**Table 7:** PLEL area under different scenarios.

Safety level	1990		2000		2010		2020	
	Area (hm²)	Proportion (%)	Area (hm²)	Proportion (%)	Area (hm²)	Proportion (%)	Area (hm²)	Proportion (%)
I	269175	36.75	323875	44.22	362100	49.44	504200	68.84
II	232700	31.77	213825	29.19	169550	23.15	168150	22.96
III	186675	25.49	158675	21.66	158075	21.58	37000	5.05
IV	34050	4.65	30950	4.23	30900	4.22	18025	2.46
V	9850	1.34	5125	0.7	11825	1.61	5075	0.69

**Fig 8:**
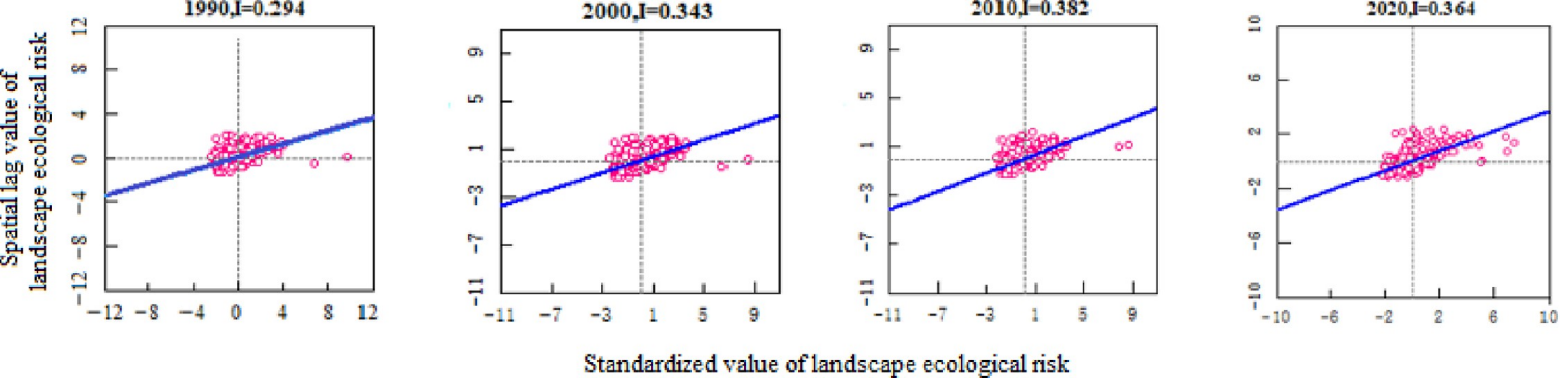
Scatter plot of landscape ecological risk I of Bole from 1900 to 2020.

From Table 7 and Fig 6, it can be seen that Bole is mainly composed of level I, II and III safe zones, accounting for more than 90% of the total area. The average landscape ecological risk values for the four periods are 0.007, 0.0066, 0.0064 and 0.0058 respectively. The landscape ecological risk shows a downward trend and the landscape ecological security is gradually improving. From Fig 8, it can be seen that the Moran’s index (I) of landscape ecological risk in Bole for the four periods are 0.294, 0.343, 0.382 and 0.364 respectively, all showing significant spatial autocorrelation (P<0.01), and the overall trend is upward, indicating that the distribution of landscape ecological risk values is becoming more concentrated in space. From Fig 6, it can be seen that the ecological security of Bole has significantly improved in 2020, with a total proportion of level I and II safe zones accounting for 91.8%, and the level III safe zone decreasing by 121075hm² compared with 2010. Overall, level IV and V safe zones are mainly concentrated in the northern, southern, and central urban areas of Bole, accounting for a total proportion between 3.15% and 5.99%. In the north and south areas, FEL and GEL are interlaced with each other, with high landscape fragmentation; in the concentrated urban areas, due to the complex interweaving of production-living land use, the landscape fragmentation degree is high and the separation degree is significantly increased. Level I safe zones are mainly distributed in the central and southwestern parts of Bole. Most of the APL in the central part presents a concentrated contiguous pattern, while the southwestern part is Sayram Lake and GEL with strong landscape stability and less external interference. Level III safe zones from 1990 to 2010 and level II safe zones in 2020 are mostly distributed in OEL with obvious contour boundaries. This type of land use is mainly characterized by Gobi and desert landscapes, so landscape ecological security is not very high. According to Fig 6, from 1990 to 2020, level II safe zones have been transferred to level I safe zones; level III safe zones have been transferred to level I and II safe zones; level IV and V safe zones have been transferred to level III and IV safe zones. With the development of regional security towards a better direction, it indicates that the overall ecological environment of Bole has improved and landscape ecological security has developed towards a benign direction.

With the increase of high security level area in the region, it indicates that the overall ecological environment of Bole City has improved over the past 30 years, and the regional landscape ecological security is developing towards a better direction.

#### Coupling characteristics analysis of PLEL landscape ecological security

**Fig 9:**
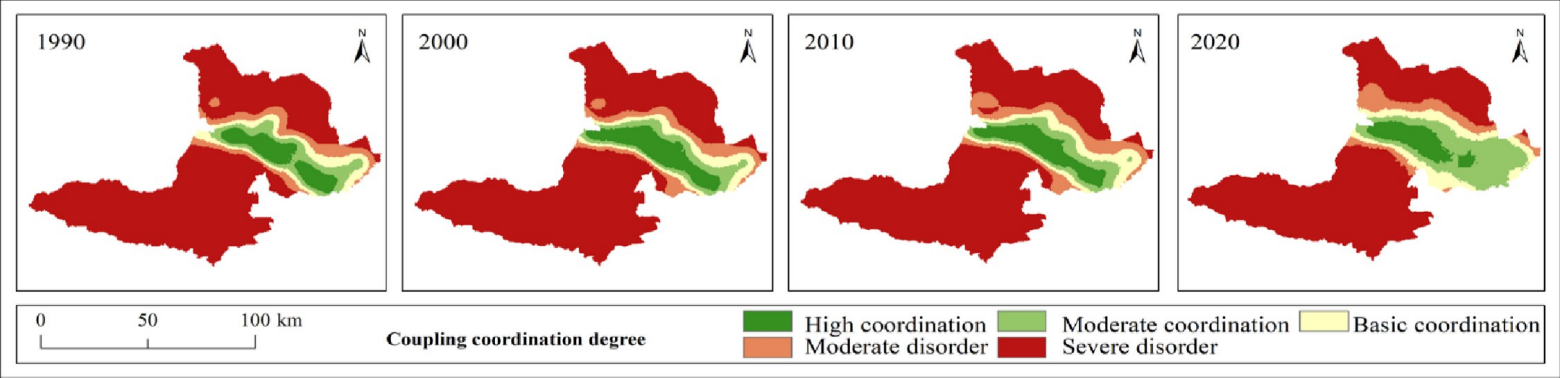
1990–2020 coupling coordination degree of landscape ecological security level distribution pattern. Republished from [57] under a CC BY license, with permission from Resource and environmental science data registration and publishing system, 2023.

**Fig 10:**
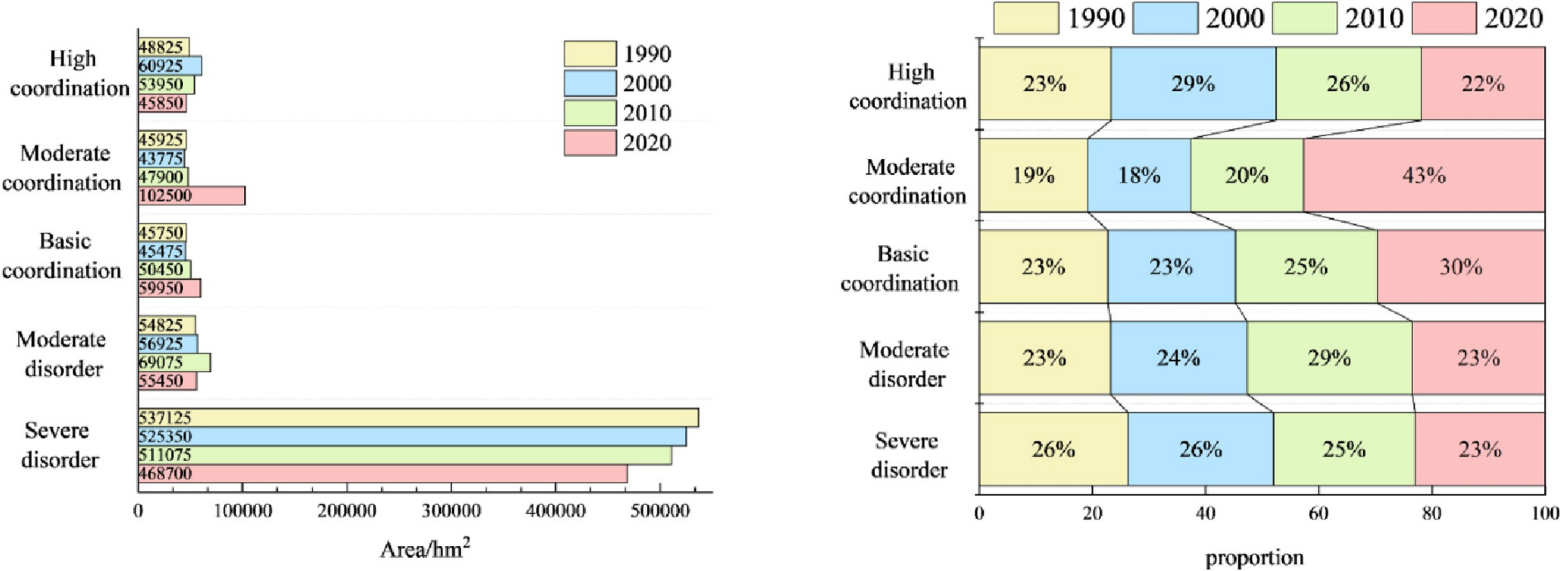
Area and proportion of coupling coordination degree of landscape ecological security.

It is of great significance to explore the coupling characteristics of PLEL for the study of regional ecosystem security. In order to further analyze the spatial coupling coordination degree of landscape ecological security in the study area, based on the landscape ecological risk value of the study area, with the help of coupling coordination degree model, using quantitative analysis method, combined with the actual situation of the study area, the coupling coordination degree is divided into five levels: severe disorder (D≤0.0093), moderate disorder (0.0093<D≤0.017), basic coordination (0.017<D≤0.025), moderate coordination (0.025<D≤0.034), and high coordination (D>0.034). Finally, the distribution map of coupling coordination degree level of landscape ecological security in Bole (Fig 9) and the area and proportion map of coupling coordination degree level of landscape ecological security (Fig 10) are obtained.

The overall coupling and coordination degree of PLEL landscape ecology security in Bole presents a distribution characteristic of “high in the middle and low in the north and south” (Fig 9). The highly coordinated area, moderately coordinated area, and basically coordinated area are mainly distributed in the agricultural and urban development areas near the Bortala River Basin in the middle of Bole. This area is suitable for human production and living activities due to its low altitude, flat terrain, and wide distribution of water sources. Therefore, PLEL is distributed here, and the overall coupling coordination level is relatively high. Due to the high altitude and less water sources on both sides of Bole, human development is less, and production land and living land are relatively rare and scattered. Therefore, the overall layout of PLEL is mostly ecological land, so the coupling coordination level is relatively poor. From 1990 to 2020, the area of basically coordinated, moderately coordinated, and highly coordinated areas has been expanding continuously. On the one hand, it shows that Bole has developed rapidly in urbanization and land reclamation in the past 30 years. On the other hand, it shows that the spatial layout of PLEL in Bole is more reasonable during this process, which is conducive to Bole’s sustainable development. Combined with Fig 10, the area of highly coordinated areas is decreasing. The area in 2020 is the smallest, which is 45850hm². The proportion of highly coordinated areas in four periods is 22%. It shows that with the development of urbanization in Bole, towns have squeezed out other land types around them, and the highly coupled PLEL layout has become unbalanced; The area of moderately coordinated areas increased by 56575hm², accounting for 43% of four periods. The proportion of basically coordinated areas was 30% in four periods. It shows that with the development of urbanization and agricultural production land expanding outwardly, more regions have realized PLEL distribution. The area of basically coordinated areas and moderately coordinated areas has been increasing continuously. Correspondingly, from 1990 to 2020, the area of seriously imbalanced areas in Bole has been gradually decreasing from 537125hm² in 1990 to 468700hm² in 2020. It decreased by 68425hm², indicating that the coupling coordination degree of PLEL in Bole is constantly improving.

In summary, the coupling coordination of PLEL in Bole is constantly improving.

### Analysis of landscape ecological security changes and coupling characteristics under multiple scenarios

#### Analysis of landscape ecological security changes under multiple scenarios

**Fig 11:**
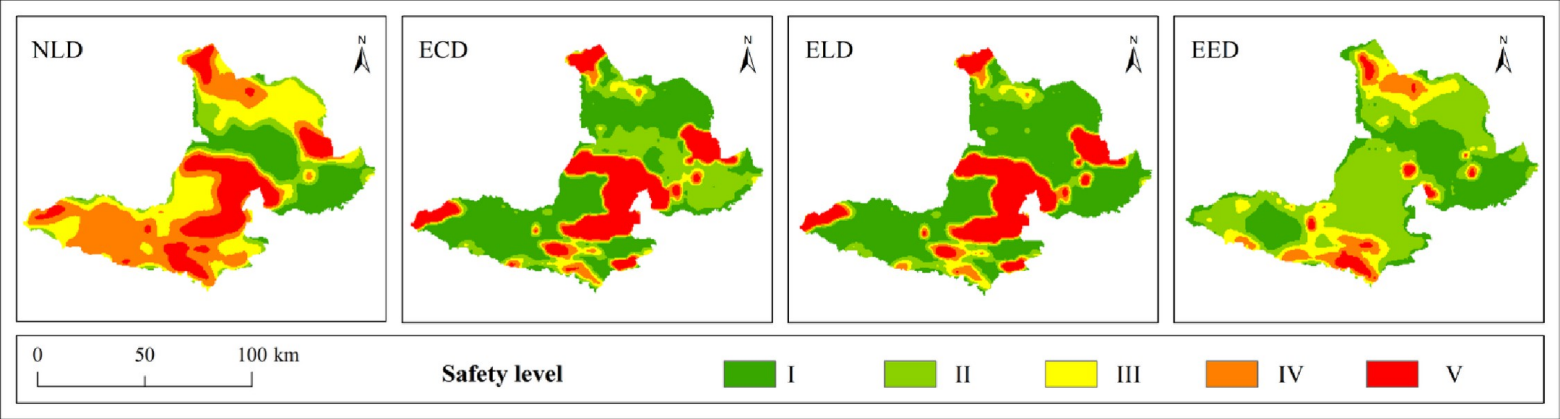
Spatial distribution pattern of landscape ecological security level under different scenarios. Republished from [57] under a CC BY license, with permission from Resource and environmental science data registration and publishing system, 2023.

**Fig 12:**
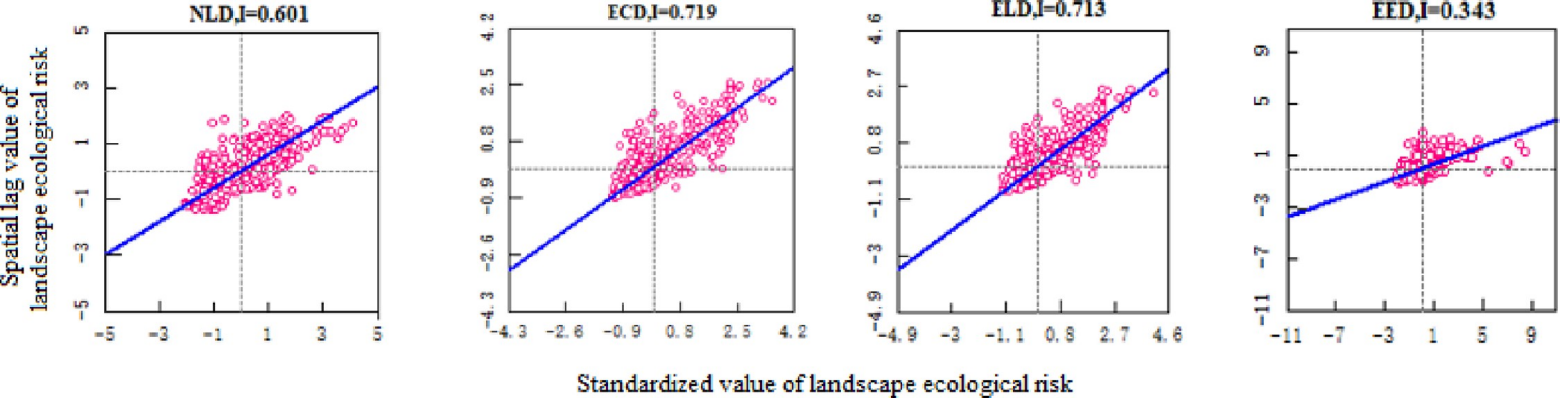
Scatter plot of landscape ecological risk I in Bole under NLD-EED scenario.

**Fig 13:**
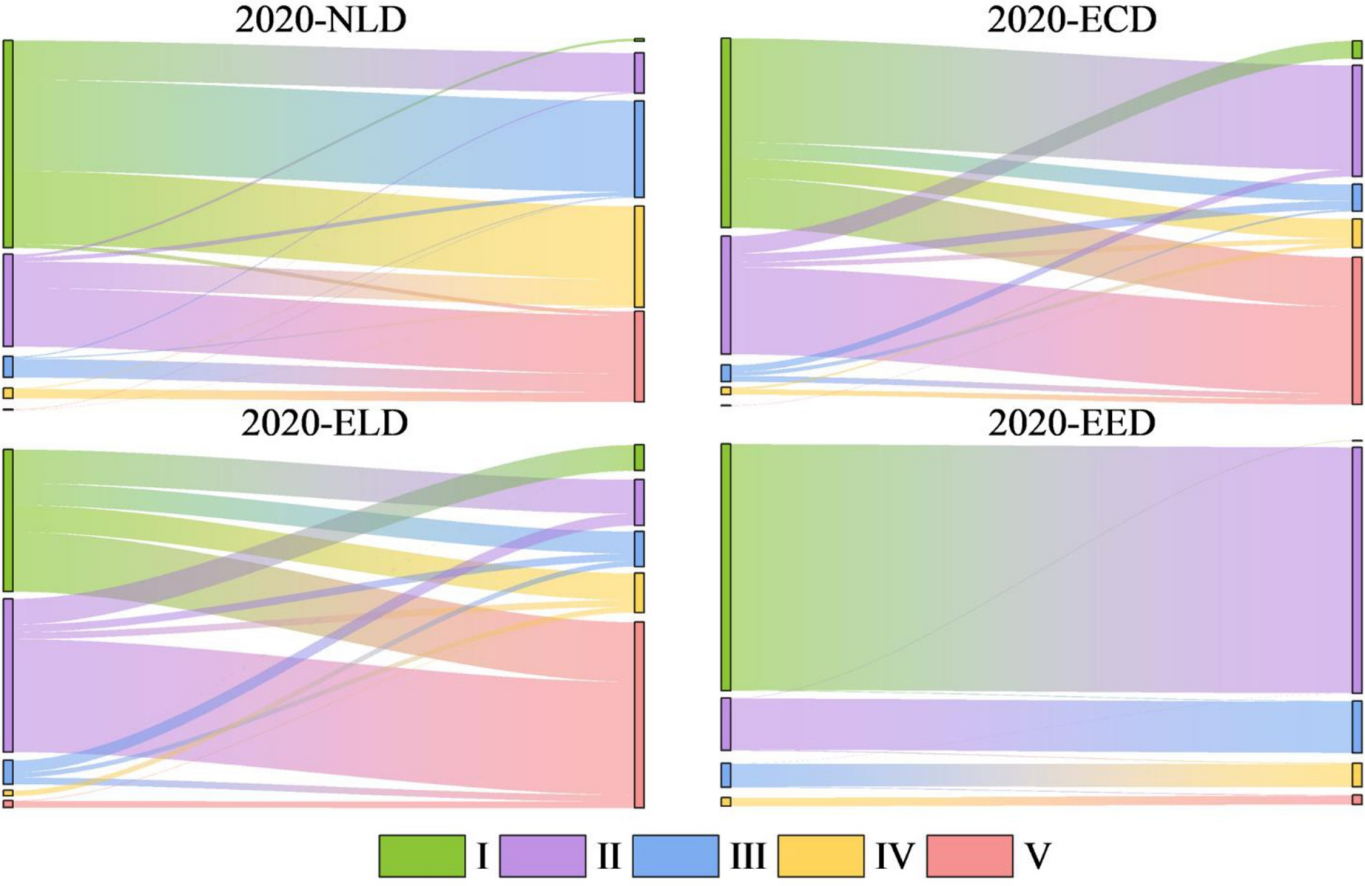
Sankey map of landscape ecological security transfer under different scenarios.

Based on the land use prediction results, the landscape ecological security level area, proportion and level distribution under four scenarios in Bole in 2030 were obtained by using the landscape ecological security index interpolation processing (Table 8, Fig 11). From Fig 12, it can be seen that the Moran index (I) of landscape ecological risk from NLD scenario to EED scenario are 0.601, 0.719, 0.713 and 0.343 respectively. The Moran index values under NLD, ECD and ELD scenarios are relatively large. Compared with the EED scenario, the landscape ecological risk values under these three scenarios have a stronger positive correlation aggregation distribution in space. The average landscape ecological risk values under these four scenarios are 0.0093, 0.0088, 0.0084 and 0.0066 respectively. Among them, the landscape ecological risk under the EED scenario is the smallest and the landscape ecological security is relatively good. However, compared to 2020, the number of different land use patches simulated by the PLUS model has increased, and their distribution has become more dispersed. This has led to a reduction in landscape connectivity. The average landscape ecological risk values under the four scenarios are higher than those in 2020, and the overall landscape ecological risk of Bole has increased. The NLD scenario is mainly composed of level III, IV and V safety zones with a total area of 500500hm² and a proportion of 68.33%. The distribution area is mainly in the north and south of Bole. Due to its fragile ecological environment, OEL mainly shows V safety zone; FEL and Sayram Lake mainly show IV safety zone; GEL mainly shows III safety zone. The ECD and ELD scenarios are mainly composed of level I, II and V safety zones with a total area of 646600hm² and 649625hm² respectively, accounting for 87.92% and 88.32% respectively; Level I and II safety zones are mainly distributed in FEL, GEL as well as production and living land; The distribution of level V safety zones is still mainly OEL. The EED scenario is mainly composed of level I and II safety zones with a total proportion of 83.93%. The area proportion of level IV and V safety zones is relatively small compared with other three scenarios, accounting for only 6.41%, indicating that the landscape ecological security of Bole is relatively good under the EED scenario. According to Fig 13, it can be seen that the overall landscape ecological security levels under different scenarios are moving towards more unsafe direction, indicating that the landscape ecological security under these four scenarios has become worse compared with that in 2020. In the NLD scenario, more level I safety zones are transferred to level III and IV safety zones, while more level II safety zones are transferred to level V safety zones; In the ECD and ELD scenarios, level II safety zones tend to shift more towards level V safety zones; In the EED scenario, more level I safety zones are transferred to level II safety zones, and less are transferred to level V safety zones. Overall, the differentiation of landscape ecological security in Bole has a useful land type orientation, with the distribution of level IV and V safety zones being particularly prominent; The proportion of level V safety zone area under NLD, ECD, and ELD scenarios ranges from 20.7% to 21.76%, which is about 10 times higher than that under EED scenarios.

**Table 8:** Area and proportion of landscape ecological security level under different scenarios.

Safety level	NLD		ECD		ELD		EED	
	Area (hm²)	Proportion (%)	Area (hm²)	Proportion (%)	Area (hm²)	Proportion (%)	Area (hm²)	Proportion (%)
I	152350	20.8	325975	44.32	412150	56.27	261350	35.35
II	79600	10.87	162075	22.04	83150	11.35	359100	48.57
III	167025	22.8	48275	6.56	45875	6.26	71450	9.66
IV	174100	23.77	40625	5.52	39650	5.41	32925	4.45
V	159375	21.76	158550	21.56	151625	20.7	14450	1.95

In summary, although the landscape ecological security under the four scenarios has decreased compared to 2020, the landscape ecological security under the EED scenario performs relatively well among the four scenarios. Therefore, simulating Bole according to the EED scenario is the best solution.

#### Coupling characteristics analysis of PLEL landscape ecological security under multiple scenarios

**Fig 14:**
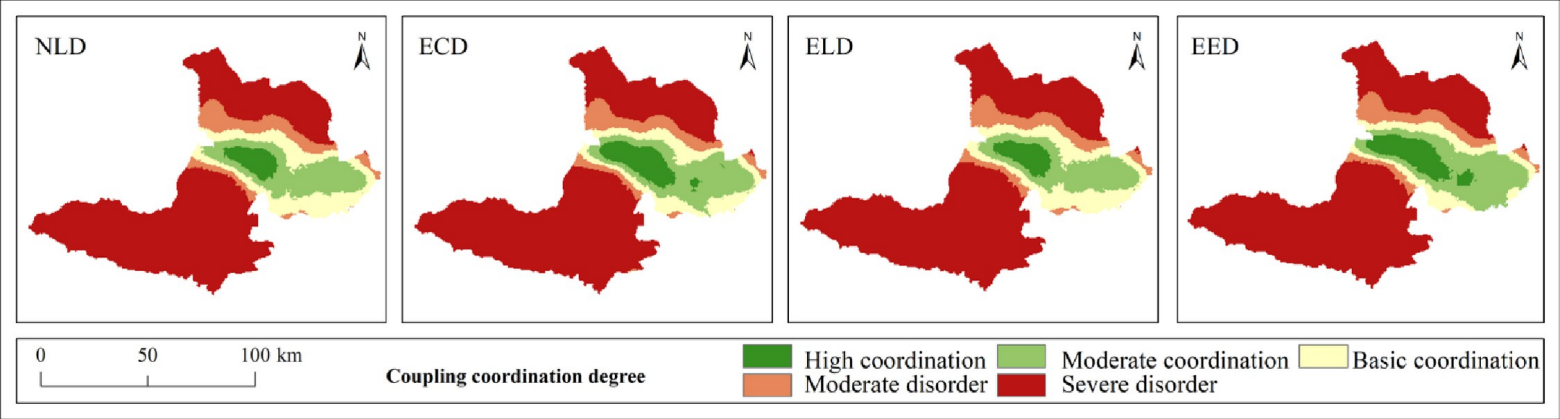
Distribution pattern of coupling coordination degree of landscape ecological security under different scenarios. Republished from [57] under a CC BY license, with permission from Resource and environmental science data registration and publishing system, 2023.

**Fig 15:**
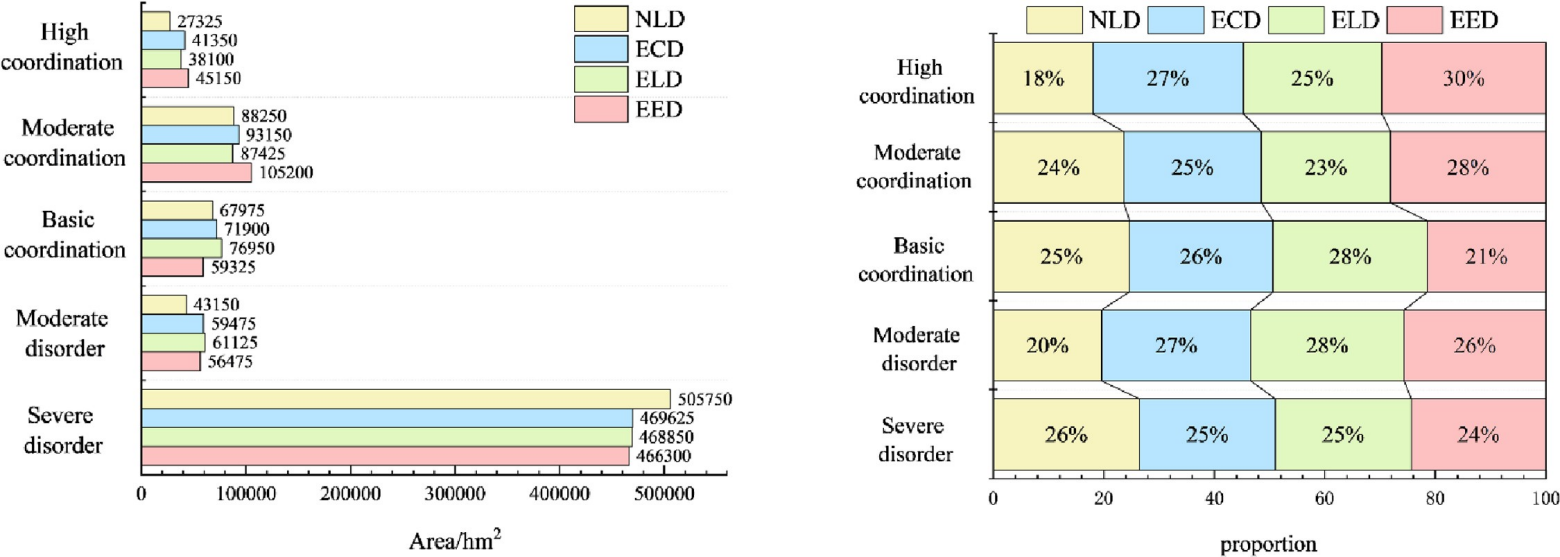
Area and proportion of coupling coordination degree of landscape ecological security under different scenarios.

According to the distribution of PLEL landscape ecological security coupling coordination degree level in the four scenarios of Bole, it can be seen from Figs 14 and 15 that overall, the distribution of PLEL landscape ecological security coupling coordination degree level in the four scenarios of Bole still shows the characteristics of “high in the middle and low in the north and south”. Compared with the other three scenarios, the area of high coordination in NLD scenario is the smallest, which is 27325hm², accounting for 18% of the total area in the four scenarios. The area of serious imbalance is the largest, which is 505750hm². The average area of serious imbalance in the other three scenarios is 468258.3hm². Therefore, NLD scenario has the worst performance in terms of PLEL landscape ecological security coupling coordination level. Due to its focus on economic development and significant expansion of urban and cultivated land, ECD scenario has higher areas of high coordination and moderate coordination, accounting for 27% and 25% respectively among the four scenarios. The basic coordination area is largest in ELD scenario, which is 76950hm², accounting for 28% among the four scenarios. The landscape ecological security coupling coordination level under ECD and ELD scenarios is in the middle position among the four scenarios. The PLEL landscape ecological security coupling coordination level under EED scenario performs best. The areas of high coordination and moderate coordination are largest among the four scenarios, which are 45151hm² and 105200hm² respectively, accounting for 30% and 28% respectively. This indicates that under EED scenario, Bole’s PLEL distribution is better.

Therefore, among the four scenarios, the PLEL distribution in Bole is better under the EED scenario.

## Discuss

### Application of the model

Using the GMOP model, the constraints of different land use types in the future can be set, and the required land use quantities for each scenario can be calculated based on the objective function of different scenarios. This provides a specific reference value for the prediction of the PLUS model. Based on the 30m×30m land use data, the predicted changes in land use have a high level of simulation accuracy in terms of structure and distribution patterns. In the NLD and ECD scenarios, with the development of the economy, the area of production and living land continues to increase, while the area of ecological land decreases overall. In the ELD scenario, the increase in production and living land area is very slow, and there is a greater inclination towards protecting ecological land, therefore, the reduction in ecological land area is the smallest among the four scenarios. In the EED scenario, there is a balanced development of eco-economy, and the allocation of land use patterns is more reasonable: economic development is balanced with ecological environment protection, the expansion of production and living land slows down, and the growth of ecological land area is slow.

Based on previous studies in Bortala Mongolian Autonomous Prefecture, the study shows that under all scenarios, the area of arable land is increasing, while the area of grassland and unused land is decreasing [58]. Additionally, in the NLD scenario, the area of forest, grassland, and unused land in Bortala Mongolian Autonomous Prefecture decreases, while the area of other land types increases, which is consistent with previous studies [59]. Unlike traditional ecological security assessment, regional ecological security assessment pays more attention to the spatial coupling relationship among various elements. Based on the PLEL perspective, this study couples the PLEL concept with regional ecological security for coordinated analysis, avoiding treating PLEL as an isolated entity and expanding the research level of PLEL in the arid Northwest region. In mainland cities, rapid economic development, large urban scale, and relatively concentrated industries result in serious coordination imbalance in urban clusters due to the encroachment of construction land on other land uses [3]. However, in arid urban areas, due to factors such as natural conditions, population, and economic development, the urban scale is not large, and the industries are relatively dispersed, which leads to a high level of coupling and moderate coupling of landscape ecological security on oases with water supply. In order to avoid serious coordination imbalance like urban clusters in mainland cities, it is necessary to reasonably plan and optimize the PLEL of arid oasis towns in a timely manner, and strive to maintain a good level of regional landscape ecological security and coupling coordination.

### Distribution characteristics and policy opinions of landscape ecological security

**Fig 16:**
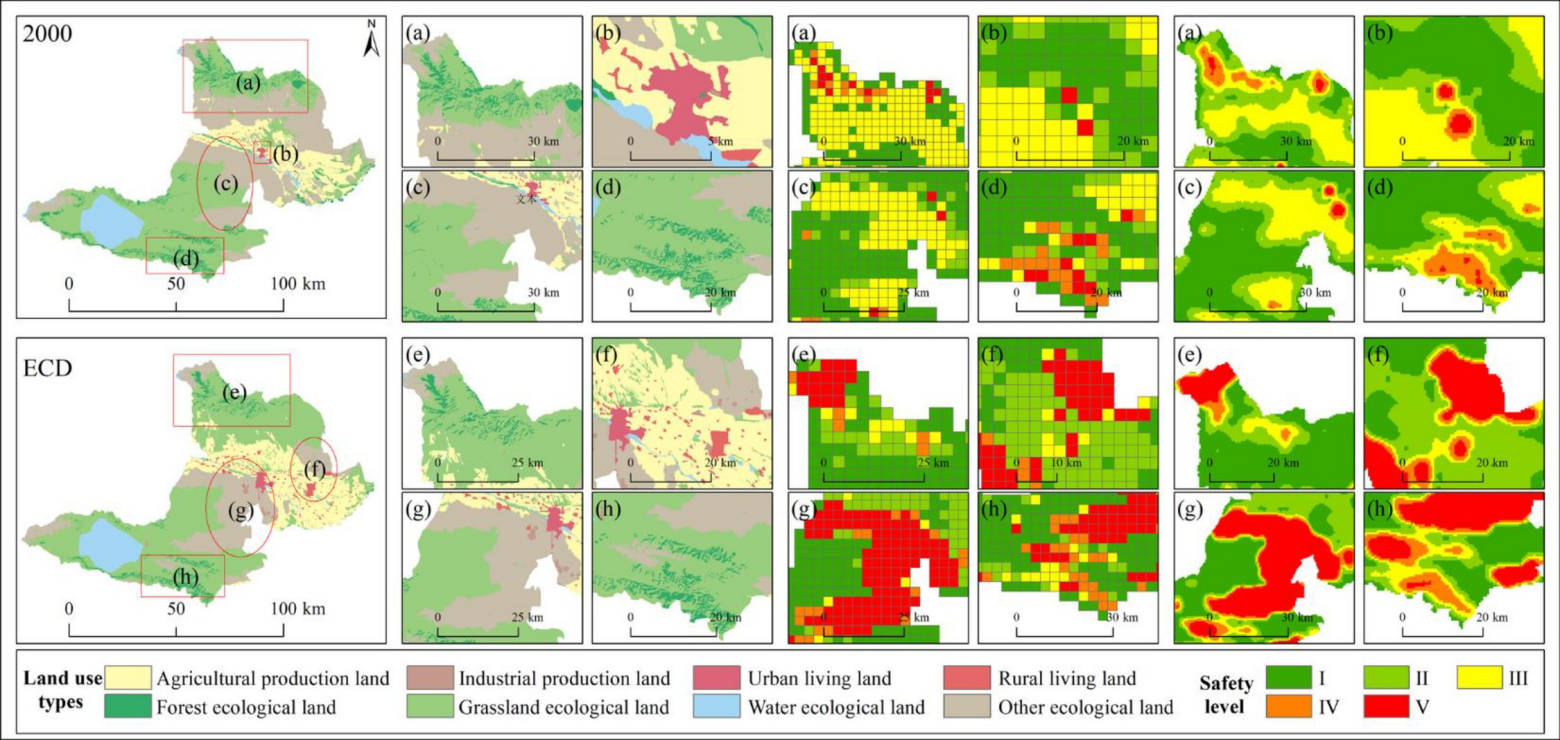
PLEL distribution and local landscape ecological risk map under 2000 and ECD scenarios. Republished from [57] under a CC BY license, with permission from Resource and environmental science data registration and publishing system, 2023.

The distribution of landscape ecological security has obvious land use type orientation characteristics. From Fig 16, it can be seen that the areas with poor landscape ecological security are mainly distributed in the OEL, FEL and GEL interlaced zone as well as ULL and RLL.

In response to OEL, FEL and GEL, it is necessary to strengthen the protection of these ecological lands. OEL itself has poor ecological environment quality, high ecological fragility and sensitivity. Therefore, in the development and utilization of OEL, the government should make plans in advance, strictly control the scale and boundary of land development, and avoid arbitrary development that may cause damage to OEL. The area of FEL is decreasing. In response to this, the government should strictly control the development of FEL, strengthen the protection of FEL, establish a forest red line, improve the tree species structure and forest quality of FEL, and strictly prevent forest fires to prevent disturbances to the landscape ecological system caused by forest fires.

For ULL and RLL, on the one hand, it is necessary to scientifically layout PLEL, strictly observe the three control lines of ecological protection red line, permanent basic farmland and urban development boundary, do a good job in urban land space planning, promote green development of urban and rural construction, strengthen the overall protection and restoration of fragmented cultivated land, forest land and shrub land in edge areas caused by urban expansion. On the other hand, we should steadily promote rural land system reform, promote urban-rural integrated planning and design, promote coordinated development of urban-rural industries. Coordinate the relationship between industrial development, urban development and ecological environment, adjust economic structure and plan industrial development. Establish a hierarchical and regional coordinated control mechanism based on natural resource carrying capacity and ecological environment capacity to reasonably determine the density and intensity of urban development and construction.

**Fig 17:**
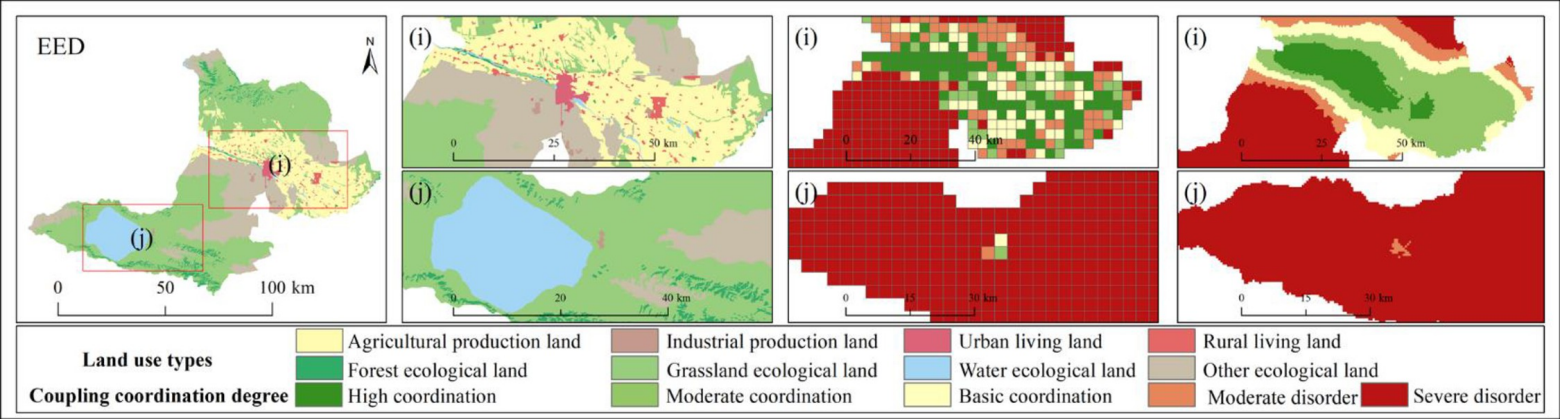
PLEL distribution and local landscape ecological coupling coordination under EED scenario. Republished from [57] under a CC BY license, with permission from Resource and environmental science data registration and publishing system, 2023.

The continuous reclamation of APL will consume a large amount of water resources in the Bortala River Basin, which will reduce the inflow of Ebinur Lake water and damage the overall ecological security of the Ebinur Lake Basin. Therefore, it is necessary to combine local water resources to determine the cultivated land based on water volume, and to carry out fragmented treatment of farmland, establish intensive, mechanized and high-standard farmland, and vigorously develop water-saving irrigation agriculture. Reasonably and effectively use the tourism resources of Sayram Lake to create a tourism industry and scenic park based on Sayram Lake, increase regional income, and reasonably layout PLEL centered on Sayram Lake (Fig 17).

In the selection of future urban development scenarios, we should not only consider the relevant policies and future target planning of China’s urban development, but also combine the optimization of the ecological security pattern in the research area under various scenario modes, and realize high-quality and sustainable development of local economy, society and ecological environment.

### Restrictions

With the development of socio-economics, the factors influencing regional ecological security are becoming increasingly complex. However, this study only uses land-use data to construct a landscape ecological risk model, lacking consideration of multiple sources of remote sensing and socio-economic factors. In calculating landscape ecological risk, disturbance and vulnerability are selected to build an evaluation model for landscape ecological security. However, the vulnerability of landscape types is relative and possesses complexity and uncertainty. In this study, the vulnerability values are subjectively assigned and lack objectivity. In future work, a more comprehensive urban ecological security evaluation system could be constructed from various perspectives, such as natural factors, socio-economic factors, ecosystem services, and policy factors. This would facilitate an overall evaluation of urban ecological security. The calculation of vulnerability in landscape patterns needs to consider both subjective and objective factors. Additionally, it is necessary to further refine the land-use classification system and the PLEL spatial index system, and construct reasonable classification criteria based on research goals and the actual conditions of the study area. This is also an important direction for future research on regional landscape ecological security assessment.

## Conclusions

1. From 1990 to 2020, the main land uses in Bole were GEL and OEL, accounting for 48.89% and 20.62% respectively. The areas of production land and living land have been continuously increasing, indicating the ongoing industrialization, urbanization, and land reclamation in Bole over the past 30 years.
2. Under the NLD and ECD scenarios, both production land and living land have shown significant growth, with a total increase of 11,325.78 hm² and 11,576.52 hm² respectively. This has had a certain impact on the surrounding ecological land, affecting the ecological balance. In the ELD scenario, the changes in IPL and living land are minimal. While this is favorable for ecological conservation, it is not conducive to urban economic development. Under the EED scenario, the development of various types of land is relatively balanced, effectively balancing ecological protection and economic development.
3. The average landscape ecological risk value in Bole has been decreasing from 1990 to 2020, indicating a positive trend towards regional security and gradual improvement in landscape ecological security. The overall area of coordinated development zones in Bole has been increasing, but the area of highly coordinated development zones has been decreasing, indicating an imbalance in the distribution of production and living land.
4. In 2030, the landscape ecological risk values under all four scenarios are higher compared to 2020. However, the EED scenario has the smallest average landscape ecological risk value, indicating a relatively better landscape ecological security. Under the EED scenario, the areas of highly coordinated development zones and moderately coordinated development zones are the largest, showing the best coupling coordination level between PLEL distribution and landscape ecological security.

This study analyzed the changes in various types of land use in Bole from 1990 to 2020 and 2030 through the PLEL perspective, exploring the coupling and coordination characteristics of landscape ecological security and PLEL at different stages, providing scientific basis and decision-making support for land resource utilization and ecological construction in Bole. However, the above research only evaluates landscape ecological security and analyzes coupling coordination based on landscape pattern. In the future, it can enrich evaluation indicators and build a comprehensive PLEL classification system, thereby comprehensively evaluating urban landscape ecological security at multiple levels and dimensions.
